# Children aged 5–13 years show adult-like disgust avoidance, but not proto-nausea

**DOI:** 10.1177/23982128241279616

**Published:** 2024-09-06

**Authors:** Sameer N.B. Alladin, Dani Berry, Evgeniya Anisimova, Ruth Judson, Poppy Whittaker, Edwin S. Dalmaijer

**Affiliations:** School of Psychological Science, University of Bristol, Bristol, UK

**Keywords:** Development, disgust, electrogastrography, gut–brain axis, interoception

## Abstract

Disgust is a vital emotion in the avoidance of illness. Human adults across cultures show disgust towards sources of potential contamination or pathogens, and elect to avoid their ingestion or even to look at them. Stomach rhythms appear to play an important role: disgust reduces normogastric power, and the pharmacological normalisation of gastric state reduces disgust avoidance. Human children are remarkably slow to develop disgust as measured by self-report and facial expressions. Here, we investigate whether disgust-induced avoidance (measured using eye tracking) and changes in gastric rhythm (measured using electrogastrography) exist in children aged 5 to 13 years (*N* = 45). We found that children in this bracket showed oculomotor avoidance of disgusting stimuli in a preferential-looking task, similar to adult samples in previous research. However, in contrast to adult samples in previous research, children did not show an attenuation in normogastric power. These findings could suggest that avoidance behaviour precedes gastric involvement during disgust. This would support the idea that children initially respond to parental modelling: parents set (and enforce) the social norm of disgust avoidance, and children initially conform and only later do they internalise disgust as an interoceptive signal. Alternatively, the employed stimuli could have been potent enough to induce oculomotor avoidance, but not a gastric response. Research is slim in this area, and future work should focus on elucidating the role of the stomach in disgust, and on longitudinal studies of disgust development from childhood to adolescence.

## Introduction

Disgust is considered one of the core emotions that exist cross-culturally and universally (Ekman and Friesen, 1971). Importantly, the emotion of disgust has an adaptive function, having evolved as a mechanism to prevent or slow the ingestion and to motivate the expulsion of potentially pathogen-rich substances (Angyal, 1941; Rozin and Fallon, 1987). As a result, humans show strong and unyielding avoidance of disgusting stimuli, even if stimuli are known to be fake (Rozin et al., 1986) or photographs on a screen (Dalmaijer et al., 2021).

Disgust is also reflected in a distinct facial response that positions the nostrils, mouth, and salivary glands that could serve to limit the potential intake of pathogens and contaminants (De Jong et al., 2011; Ekman and Friesen, 1971; Olatunji et al., 2008; Rozin and Fallon, 1987; Shenhav and Mendes, 2014; Van Overveld et al., 2009). Further down the gastrointestinal tract, there is a stomach response to core disgust elicitors (e.g. bodily effluvia): normogastric rhythms are attenuated (Shenhav and Mendes, 2014), with relative increases in tachygastria (Harrison et al., 2010). Because of its resemblance to the gastric physiology of nausea, we have previously described this phenomenon as ‘proto-nausea’ (Alladin et al., 2023).

Evidence from the causal involvement of the stomach in disgust comes from a drug study in which the administration of domperidone (but not placebo) combined with encouraged exposure to disgusting stimuli reduced the avoidance of those stimuli (Nord et al., 2021). In human adults, there thus seems to be a direct link between gastric dysrhythmia and disgust avoidance. This aligns with theoretical frameworks, which describe the disgust’s oral origins (Angyal, 1941) and put nausea at the core of many of its processes in humans and other animals (Case and Stevenson, 2024; Stevenson et al., 2019).

Curiously, and to parents perhaps worryingly, children do not show strong disgust avoidance. Parental reports of children responding to disgust elicitors only plateau at age 7 years, and the same is true for their self-report and facial expressions to core disgust elicitors (Stevenson et al., 2010). This is remarkable, because children are uniquely susceptible to illness between ages 1 to 5, when maternal antibodies have worn off and the innate immune system is still immature (Simon et al., 2015). They would thus greatly benefit from the avoidance of pathogens that a sense of disgust would encourage. Some even argue that its late onset draws into question disgust’s adaptive role in disease avoidance (Rottman, 2014).

However, children do not necessarily have to rely on internal signals: children generally follow social norms, and even enforce them between each other (Schmidt et al., 2012). Hence, when parents set the social norm of disgust-avoidant behaviour (‘*Do NOT stick that in your mouth!*’), children could follow even in the absence of strong feelings of disgust. In addition to parental modelling, disgust sensitivity is thought to be shaped by social norms in, for example, peer structures and societies (Dalmaijer and Armstrong, 2020; Tybur et al., 2013, 2018). In this scenario, children would not necessarily show adult-like proto-nausea to typical disgust elicitors, but they would show behavioural avoidance.

We address this question by testing disgust avoidance and gastric reactivity during childhood, here defined as ages 5–13 years. If children indeed only show adult-like disgust sensitivity from age 7, disgust avoidance and disgust-induced proto-nausea should increase and then plateau during childhood. However, if children are only slow to internalise disgust sensitivity, avoidance behaviour should already be present uniformly across childhood (in response to the social norm), and only proto-nausea should increase with age (as the social norm is internalised).

## Methods

### Participants

Prior to data collection, ethical approval was obtained from the School of Psychological Science Research Ethics Committee at the University of Bristol (approval code: 11578). Families were recruited through a snowball procedure using social media and word of mouth. They were compensated for their travel, and with £20 for their time.

Exclusion criteria were an inability to understand information and instructions in English (for both parent/carer and children), and the inability to provide assent (children) or consent (parents/carers).

A total of 44 participants between the ages of 5 to 13 years were recruited. Two participants prematurely quit the passive-viewing task (during which electrogastrography (EGG) was recorded), leaving 42 for analysis. For the preferential-looking task, 6 participants were excluded due to device malfunction or incompatibility (e.g. due to glasses), leaving 38 for analysis. No exclusions occurred for questionnaire data. The number of complete datasets was 37, but incomplete datasets were not excluded for analyses for which sufficient data existed. For example, a correlation between proto-nausea and child self-reported disgust sensitivity could be computed for *N* = 42, a correlation between proto-nausea and disgust avoidance with *N* = 37, a correlation between disgust avoidance and child self-reported disgust sensitivity with *N* = 38, and a correlation between subscales of the questionnaire for child self-reported disgust sensitivity with *N* = 44.

### Procedure

Before their visit, participants were offered information and the opportunity to ask questions before committing. During the visit, parents were provided with an information sheet, children with verbal information (read aloud from an age-appropriate information sheet), and both were given the opportunity to ask questions. After this, parents/carers provided written informed consent, and children provided verbal assent. Throughout the session, participants were free to prematurely stop a task, questionnaire, or participation altogether.

Parents/carers were offered a set of questionnaires to complete. Children completed a preferential-looking task to measure disgust avoidance, and a passive-viewing task to measure proto-nausea. The order of tasks was counter-balanced. Tests occurred in two different spaces, which allowed concurrent testing of up to two children (when parents/carers brought more than one). See below for further details on questionnaires and tasks.

After completing both tasks, children were asked to verbally complete a questionnaire. Items were read aloud by an experimenter, and children provided a verbal response that was written down by the experimenter.

After completing (or prematurely stopping) the tasks and questionnaires, participants were provided with a debriefing that included information on the purpose and potential outcomes of the study, and another opportunity to ask any questions.

### Measures and materials

#### Stimuli

A total of 14 disgusting and 14 neutral images were selected from our previous work (Anwyl-Irvine et al., 2022; Dalmaijer et al., 2021; Nord et al., 2021) and the DIsgust RelaTed Images (DIRTI) database (Haberkamp et al., 2017). The disgusting images portrayed bodily effluvia (specifically faeces, nasal mucous, and vomit), and neutral images portrayed visually similar scenes or objects. All images were suitable for children to view.

#### Preferential-looking task

During a preferential-looking task, stimuli are shown side-by-side, and participants are free to view them according to their own preferences. For disgust, but not for other negatively or positively valenced stimuli, this typically leads to sustained relative avoidance after a brief (~1 s) initial period of approach (Armstrong et al., 2022; Dalmaijer et al., 2021). That is, on average, participants spend more time looking at neutral images presented alongside disgusting images, and this bias remains unchanged over the course of an experiment even if stimuli are frequently repeated (Dalmaijer et al., 2021).

In our implementation, 14 stimulus pairs were repeated three times, which resulted in 42 trials in total. During each trial, a fixation cross was visible for 1 s, followed by the stimulus pair for 12 s, and then an inter-trial interval with a blank screen for 3 s. Stimulus pairings were fixed, but the order of stimulus pairs was randomised.

Eye movements were recorded throughout the task using an EyeLink 1000. At task onset, this was calibrated using a 9-point grid. We quantified dwell time for either stimulus using the total number of gaze samples with pixel coordinates within the displayed stimulus, and all other samples were considered ‘other’. Disgust avoidance was quantified as the difference between dwell times for disgusting and neutral stimuli.

#### Passive-viewing task

The passive-viewing task aimed to measure proto-nausea in response to disgust elicitors. Because the normogastric rhythm is slow (1 cycle per ~20 s), we employed a block design to accurately capture power across relevant frequencies (~1–10 cycles/min). Participants were thus presented with two blocks of image presentations: disgusting and neutral. The order of blocks was randomised, as was the order of stimuli within each block. Each stimulus was presented twice. EGG was recorded throughout this task.

In each trial, a fixation dot was presented for 0.4–0.6 s, followed by the presentation of a single image for 20 s, followed by an inter-trial interval of 6.5–8.5 s. During this interval, scenes from a children’s cartoon were played in an attempt to keep participants interested.

#### Electrogastrography

EGG was recorded cutaneously, using a four-channel setup with four active electrodes, one reference, and one ground (Simonian et al., 2004). The reference electrode was placed on the skin just under the xiphoid process, and a ground electrode placed just below the costal margin on the participant’s left side. Electrode 3 was placed halfway between the reference electrode and the umbilicus, and electrode 4 was placed approximately 2 cm to the participant’s right of sensor 3. Electrode 2 was placed 2 cm away from electrode 3, at a 45-degree angle, in leftward direction from the participant’s perspective. Electrode 1 was placed a further 2 cm away from electrode 2, again at a 45-degree angle and in the participant’s leftward direction. We used 20 × 25 mm disposable solid gel Ag–AgCl electrodes with snap connector (Spes Medica, NeuroTab, ref. DENIS02025), chosen for its ease of removal without causing participant discomfort. Signals were recorded using an OpenBCI Ganglion amplifier with custom code written in Python 3.8.10, using BrainFlow 5.1.2, NumPy 1.22.0 (Harris et al., 2020), OpenCV (opencv-python) 4.6.0.66, PyGame 2.0.3, and PyGaze 0.7.3 (Dalmaijer et al., 2014).

The electrogastrogram is subtly impacted by participants’ position (Jonderko et al., 2005; Matsuura and Takada, 2019), and supine measurements are typically preferred over seated positions. Because testing in supine position is not directly compatible with a passive-viewing task, we opted to seat participants in a reclined position (using an Ikea ‘POÄNG’ armchair in children’s size). We also hoped this would reduce spontaneous movements in children during our (relatively boring) passive-viewing task, as the reclined armchair offered fewer degrees of freedom compared to an upright or swivel chair.

After debating asking participants to fast (which is typical prior to EGG for medical/diagnostic purposes), we ultimately elected not to do so because gastric myoelectrical activity could be unstable in a fasted state (Levine, 2005).

#### Self-reported disgust sensitivity

Children completed the disgust sensitivity questionnaire from the study by Stevenson et al. (2010). This was verbally administered by the researcher, who offered each of the 22 statements alongside four response options: ‘No, or unsure I’d be disgusted’, ‘Possibly I’d be disgusted’, ‘Probably I’d be disgusted’, and ‘Definitely I’d be disgusted’. We opted for this instrument over, for example, the Child Disgust Scale (Viar-Paxton et al., 2015) to be better able to compare our sample to that of Stevenson et al. (2010), in order to ensure appropriate contextualisation of our behavioural and gastric measurements.

Parents/carers completed the revised Disgust Scale (Haidt et al., 1994; Olatunji et al., 2007), with five response options (counted 0–4): ‘Strongly disagree’, ‘Mildly disagree’, ‘Neither agree nor disagree’, ‘Mildly agree’, and ‘Strongly agree’ for statements in items 1–14; and ‘Not disgusted at all’, ‘Slightly disgusted’, ‘Moderately disgusted’, ‘Very disgusted’, and ‘Extremely disgusted’ for scenarios in items 15–27.

A parent-specific addition to the Disgust Scale (Visconti, 2013) was also completed by parents/carers. This instrument contains 13 items with scenarios that occur when parenting children (e.g. being spat up on or changing nappies), and is scored on the same scale as the first half of the Disgust Scale.

Finally, parents/carers completed the Three-Domain Disgust Scale (TDDS; Tybur et al., 2009), which offers 21 questions equally spread across the three subdomains of pathogen, sexual, and moral disgust. Each question was answered on a 7-point scale, ranging from ‘Not disgusting at all’ (0) to ‘Extremely disgusting’ (6).

### Data reduction

#### Preferential-looking task

Gaze data were recorded at 1000 Hz, and fixations were extracted using EyeLink’s default algorithm. We counted fixations within the horizontal span of each stimulus image in trials in the preferential-looking task as ‘hits’ on that area of interest, and then summed the duration of such fixations to compute the total dwell time for each stimulus.

Trials with over 50% missing data were excluded. This occurs when participants blink or look away from the monitor, but can also be a consequence of poor eye-tracking quality (e.g. due to mismatch between calibration and situation, eye glasses, and so on). A total of 39 trials were removed from analysis, spread among 14 participants. This reflects 2.4% of the total number of recorded trials.

#### Passive-viewing task

Gastric data were recorded at 200 Hz at four sensor locations. For each sensor, we subtracted the mean from the signal, then employed a Hampel filter with a window size of 4000 samples (one normogastric cycle) and rejection threshold of 3, and a Butterworth filter with high pass of 0.5 cycles/min (~8.3e–3 Hz) and low pass of 10 cycles/min (~0.17 Hz). Then we computed an independent component analysis (ICA) to extract four underlying components. We computed the signal-to-noise ratio in each component as the ratio between peak power in the normogastric range (2–4 cycles/min) and the average power in the remaining frequencies. After excluding all components with a signal-to-noise ratio under 3, we reconstituted the electrogastrogram for all sensors by inverse-transforming the remaining components. In one participant, no components remained, and they were thus excluded from analyses including the electrogastrogram.

Finally, we computed power per frequency by applying a Hanning window and then using a fast Fournier transform for signal from each sensor for the neutral and disgust blocks separately. The resulting power spectra were averaged over sensors, and then used to compute mean, standard deviation, maximum, and proportion of power within each frequency band: bradygastric (0.5–2 cycles/min), normogastric (2–4 cycles/min), and tachygastric (4–10 cycles/min).

## Results

### Persistent oculomotor avoidance of disgust elicitors

**Figure 1.**
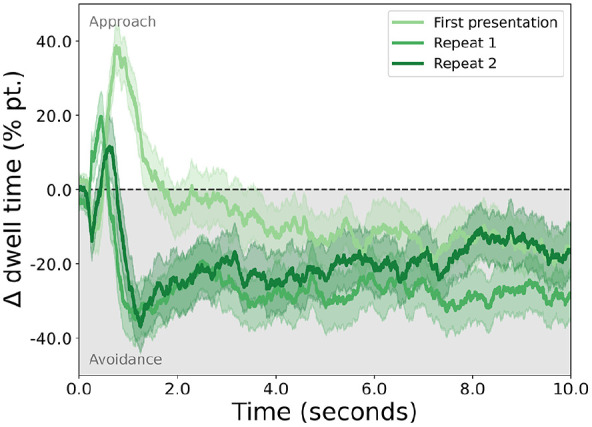
The approach or avoidance of disgusting stimuli, expressed as the difference in percentages of dwell time for the disgusting and the neutral stimuli (*y*-axis) throughout the duration of a trial (*x*-axis). Lines indicate averages (first averaged over stimulus pairs, then participants), and shaded areas standard error of the mean (between participants). On the first presentation of a stimulus pair, participants showed an initial bias towards the disgusting stimulus, followed by a sustained bias away from it. On repetitions of the same stimuli, the initial bias was attenuated, but disgust avoidance remained.

Fixations were extracted from gaze data using EyeLink’s default detection algorithm, after which all fixation durations within an area of interest (the horizontal boundaries of each stimulus) were summed to compute dwell time. When averaged over trials and participants, dwell time is biased towards the disgusting stimulus early in a trial, after which it shows stable disgust avoidance (see Figure 1). This is particularly true for the first presentation of a stimulus pair, whereas repetitions of the same stimuli lead to more pronounced avoidance. For the purposes of this study, we averaged across all presentations, as our interest here was disgust avoidance in general and not its (lack of) habituation over repeated presentations.

Dwell times were analysed with linear mixed-effects models with random effects participant number and parent/carer number (some children were siblings), and within-participant fixed effects condition (neutral and disgust) and trial number (standardised), as well as between-participant fixed effects age in months and sex (male or female; no other options were self-reported). The results of several models are reported, alongside differences in Akaike (AIC) and Bayesian Information Criterion (BIC) to quantify goodness-of-fit.

One well-fitting model [ΔAIC = 7.0, BIC = best] included only stimulus [*β* = 0.60, *Z* = 17.35, *p* < 2.22e–16], and indicated that children were more likely to dwell on the neutral than the disgusting stimulus.

Another well-fitting model [AIC = best, ΔBIC = 5.0] showed main effects of stimulus [*β* = 0.60, *Z* = 17.54, *p* < 2.22e–16] and trial [*β* = –0.10, *Z* = –3.88, *p* = 1.04e–4], and an interaction between stimulus and trial [*β* = 0.16, *Z* = 4.66, *p* = 3.16e–6]. This indicated that children were more likely to dwell on the neutral compared to the disgusting stimulus, and that this tendency increased over trials. The main effect of trial number suggested dwell times on either area of interest reduced over trials, likely due to calibration quality degradation.

Including age resulted in a worse fit [ΔAIC = 9.9, ΔBIC = 14.9], despite showing a statistically significant interaction between age and stimulus [*β* = 0.25, *Z* = 7.15, *p* = 8.68e–13] alongside main effects of stimulus [*β* = 0.60, *Z* = 17.68, *p* < 2.22e–16] and age [*β* = –0.12, *Z* = –2.31, *p* = 0.021]. If one were to subscribe to this model, it would suggest children dwelled on neutral stimuli more than disgusting stimuli, and that this increased with age. The main effect of age suggested that dwell times on either stimulus increased with age, likely due to better compliance with instructions among older children (leading to less movement and thus better calibration quality retention).

Including age and sex also resulted in a worse fit [ΔAIC = 3.0, ΔBIC = 32.1], with the model showing main effects of stimulus [*β* = 0.72, *Z* = 16.11, *p* < 2.22e–16], but not age [*β* = –0.05, *Z* = –0.78, *p* = 0.434] or sex [*β* = 0.07, *Z* = 0.73, *p* = 0.464]; and interaction effects between age and stimulus [*β* = 0.16, *Z* = 3.03, *p* = 2.48e–3], sex and stimulus [*β* = –0.29, *Z* = –4.21, *p* = 2.58e–5], and age, sex, and stimulus [*β* = 0.19, *Z* = 2.70, *p* = 6.85e–3], but not age and sex [*β* = –0.12, *Z* = –1.32, *p* = 0.188].

In sum, dwell time was lower for disgusting than for neutral stimuli, and this effect increased with trial number. The disgust-avoidance effect also increased with age, and was less strong in boys compared to girls. However, including age and sex produced worse model fits, suggesting they were not necessarily important contributors.

### No disgust-induced proto-nausea

For statistical tests, EGG signal power was reduced to peak power within the normogastric band; or to average, standard deviation, maximum, or proportion of power within each frequency band (bradygastric 0.1–2 cycles/min, normogastric 2–4 cycles/min, and tachygastric 4–10 cycles/min). These reduced data were analysed with linear mixed-effects models with random effects participant number and parent/carer number (some children were siblings), and within-participant fixed effects condition (neutral and disgust), block order (disgust–neutral and neutral–disgust), frequency band (bradygastric, normogastric, and tachygastric), as well as between-participant fixed effects using standardised scores for age in months and one of the questionnaires (specifically: child, Disgust Scale – revised, its parent extension, or the TDDS’ pathogen subscale).

For peak normogastric EGG, the best fitting model [ΔBIC = 8.7] included only an intercept, and the next-best fitting model included condition but without statistically significant relation to normogastric peak power [*β* = –0.2, *Z* = –0.36, *p* = 0.717]. This suggests that the normogastric peak was not different between the disgusting and neutral stimulus conditions.

The best-fitting model [ΔBIC = 9.1] for average power in each gastric band included only frequency band, with both normogastric [*β* = 1.30, *Z* = 16.29, *p* < 2.22e–16] and tachygastric power [*β* = 0.83, *Z* = 10.47, *p* < 2.22e–16] being higher than bradygastric power. The next-best fitting model also included block order, and the interaction between block order and gastric band. Results were highly similar, with both normogastric [*β* = 1.07, *Z* = 10.06, *p* < 2.22e–16] and tachygastric power [*β* = 0.64, *Z* = 6.05, *p* = 1.44e–9] being higher than bradygastric power, no effect of block order [*β* = –0.02, *Z* = –0.09, *p* = 0.928], and an interaction of block order and normogastric [*β* = 0.49, *Z* = 3.15, *p* = 1.63e–3] and tachygastric band [*β* = 0.41, *Z* = 2.63, *p* = 8.54e–3] compared to that of the bradygasrtic band. The same general pattern held for the standard deviation, maximum, and percentage of power within each frequency band.

These findings suggest that gastric power was different between the frequency ranges, specifically with highest power in the normogastric range (as expected). There was some evidence of an interaction with block order, but only in models that fit substantially less well overall, suggesting that the effect was minor at best. Crucially, gastric power was not impacted by the disgust manipulation, and it was not related to oculomotor disgust avoidance, self-reported disgust sensitivity, or parental disgust sensitivity.

### Correlations with and between self-report measures

**Figure 2.**
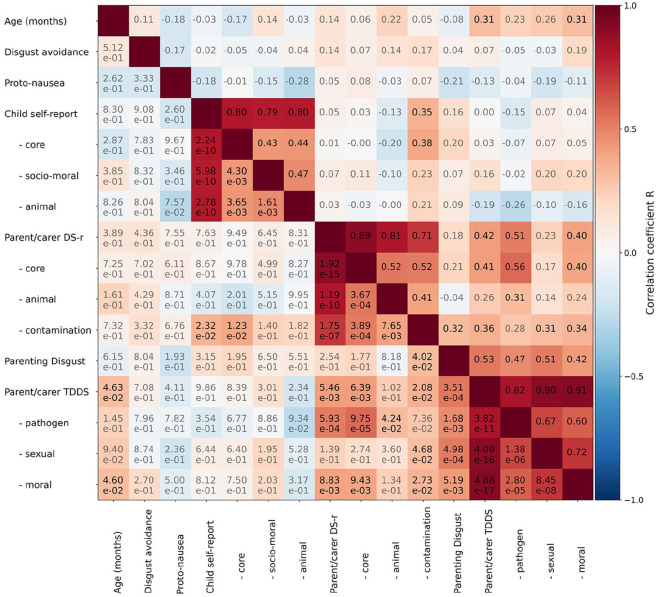
Bivariate correlations between age, disgust avoidance (measured with a preferential-looking task), gastric disgust (disgust-to-neutral ratio of peak power in the normogastric range of the electrogastrogram), and self-report measures. The top-right triangle lists the correlation coefficients, and the bottom-left triangle the associated *p*-values. Opaque lettering indicates ‘statistically significant’ outcomes (uncorrected at *α* = 0.05); other test outcomes are semi-transparent. DS-r: Disgust Scale – revised; TDDS: Three-Domain Disgust Scale.

Included self-report measures were the questions by Stevenson et al. (2010) for children; and the revised Disgust Scale (DS-R), a parenting-specific extension of the DS-r, and the TDDS for parents. Bivariate correlations between each of these self-report measures, and with age, behavioural disgust avoidance (dwell for disgust minus dwell for neutral), and gastric disgust (log of the division of normogastric peak power during disgust divided by neutral) are reported in Figure 2.

Subscales of each questionnaire correlate with each other [child: *R* = 0.43 to 0.47; parent/carer DS-r: *R* = 0.41 to 0.52; parent/carer TDDS: *R* = 0.60 to 0.72] and their combined score [child: *R* = 0.79 to 0.80; parent/carer DS-r: *R* = 0.71 to 0.80; parent/carer TDDS: *R* = 0.82 to 0.91]. Total scores between the DS-r and TDDS also correlated well [*R* = 0.42, *p* = 0.005].

Child self-report only correlated with the parent/carer contamination subscale [*R* = 0.35, *p* = 0.023], and particularly its core subscale [*R* = 0.38, *p* = 0.012]. Self-reported disgust sensitivity for parenting-associated scenarios correlated with DS-r contamination [*R* = 0.0.32, *p* = 0.040], TDDS total [*R* = 0.53, *p* = 3.51e–4], TDDS pathogen [*R* = 0.47, *p* = 0.002], TDDS sexual [*R* = 0.51, *p* = 4.98e–4], and TDDS moral [*R* = 0.42, *p* = 0.005].

No correlations were found between behavioural disgust avoidance or with gastric disgust. Care should be taken when interpreting any of these correlations due to the relatively small sample size (*N* = 44).

## Discussion

As a group, children aged 5–13 years show behavioural avoidance of disgusting stimuli as measured in a preferential-looking task. They do not show a gastric response to disgusting stimuli: not as a reduction of peak power in the normogastric range, nor as a disgust-induced difference in average, peak, variability, or proportion within each frequency band in the electrogastrogram (brady-, normo-, or tachygastric ranges). Disgust avoidance and gastric disgust did not relate to self-reported disgust sensitivity of children or their parents/carers. Correlations between self-reported disgust sensitivity were slim between children and their parents/carers, but clearly present between different questionnaires among parents/carers.

Previous work on children’s responses to disgust elicitors showed that children’s disgust to potential contaminants approaches adult-like levels from about 7 years of age, as measured with parental report, self-report, and facial expressions (Stevenson et al., 2010). Our study shows no increase in self-response, but this could be due to the low bound on our age bracket being 5 years, which left limited space for increase (i.e. children in our sample may have been at ceiling already). This was a deliberate choice, as our intention was to measure gaze and gastric responses.

### Behavioural disgust avoidance

Our study extends the existing literature by showing that behavioural avoidance as measured with a preferential-looking task is already established at 5 years of age. This aligns with a different behavioural manipulation in earlier work, which scored children’s choices in four scenarios within the core disgust domain (eating sweets from a clean potty, opening the lid to a transparent jar of larvae and potentially touching them, evaluating and potentially touching a dirty sock, and sniffing fertiliser and fermented shrimp paste), and saw adult-like behaviours towards 7 years of age (Stevenson et al., 2010).

The benefit of measuring disgust avoidance with preferential-looking was that it is an established paradigm in adults, with high reliability and good correlations to self-reported disgust and disgust sensitivity (Anwyl-Irvine et al., 2022; Armstrong et al., 2021; Dalmaijer et al., 2021). Importantly, the sustained oculomotor avoidance of stimuli is unique to disgust: fear-associated stimuli inspire a bias towards them that quickly habituates (Dalmaijer et al., 2021), negatively valenced suicide-related stimuli inspire a similar short-lived approach, and positively valenced stimuli inspire a sustained approach (Armstrong et al., 2022). Our results thus align with the cited work on children’s disgust avoidance, but also compare to a larger body of work on adults that has established non-habituating oculomotor avoidance as unique to disgust.

The above suggests that children aged 5–13 years have already established adult-like levels of behavioural disgust avoidance, including a similar early but brief bias towards disgusting stimuli (Anwyl-Irvine et al., 2022; Armstrong et al., 2022), and a lack of habituation over many trials (Dalmaijer et al., 2021).

### Proto-nausea in response to disgust elicitors

Previous studies have reported gastric dysrhythmia in response to core disgust elicitors such as bodily effluvia (Harrison et al., 2010; Shenhav and Mendes, 2014; Stern et al., 2001), and pharmacological work suggests that these stomach rhythms are causally involved in disgust avoidance (Nord et al., 2021). We have previously described this phenomenon as ‘proto-nausea’, due to its resemblance to gastric rhythms during nausea (Alladin et al., 2023).

In children aged 5–13 years, we did not find any evidence of proto-nausea. This is despite computing a variety of potential indices of gastric responses, including not only peak normogastric power, but also the average, maximum, variance, and percentage of power among each of the typically analysed bradygastric, normogastric, and tachygastric bands.

If this is a true null effect, it could mean that children first learn to express disgust (through avoidance) according to a social norm. Over time, this is internalised as an interoceptive response in the form of proto-nausea.

An alternative explanation is that our stimuli failed to induce a strong enough disgust response. It has been argued that stimuli that represent disgust elicitors (e.g. images, vignettes, or questionnaire items) produce a simulated form of disgust without the bodily threat that true elicitors pose (Stevenson et al., 2019). Empirically, simulated stimuli have been shown to induce physiological responses, for example, saliva production in response to vignettes (Van Overveld et al., 2009), and gastric effects of disgusting images (Meissner et al., 2011; Zhou et al., 2004) or videos (Shenhav and Mendes, 2014). However, it could nevertheless be argued that our specific stimuli were too weak or that children are less susceptible to simulated disgust.

### Limitations

Due to the nature of our sample, we have had to make several adaptations to our tasks. Specifically, we used stimuli that were appropriate for use in children, which limited their valence. That said, the very same stimuli were potent enough to induce a strong behavioural avoidance in our preferential-looking task, so they did inspire a level of disgust.

Another potential dampening factor was that we interleaved the presentation of stimuli with a children’s cartoon. This encouraged children to continue the passive-viewing task during which EGG was recorded; a necessity in an age group that is susceptible to boredom. The alternative solution of reducing block length was not feasible, because the slow nature of gastric rhythms (around 3 cycles/min or 0.05 Hz) requires a long sampling time for accurate measurement. As a consequence, one could argue that children might have not experienced a sufficient level of disgust throughout the experiment to provoke a sufficient stomach response.

While the human disgust-avoidance response does not habituate in the short term (Dalmaijer et al., 2021), it could reduce with long-term exposure (Edgar et al., 2024). For parents, it is frequently necessary to engage with bodily effluvia (e.g. during nappy changing and when children are sick), which is not usually true for their offspring. Parents might thus be in a state of long-term habituation, whereas their children are not. This could have blunted correlations between carers’ self-reported disgust sensitivity and disgust-related measurements in their children.

## Conclusion

Children aged 5–13 years show adult-like behavioural avoidance of core disgust elicitors, which suggests they have either internalised an adult sense of disgust, or that they are abiding by adult-set social norms around such disgust elicitors. A tempting interpretation of the absence of proto-nausea is that children have not fully internalised the social norm of disgust avoidance, and have yet to fully develop the typical gastric response to disgust elicitors. An alternative interpretation is that behavioural avoidance and proto-nausea require different levels of disgust, and that our stimuli were disgusting enough to avoid but not enough to induce a gastric response.
